# Alternative NADH dehydrogenase extends lifespan and increases resistance to xenobiotics in *Drosophila*

**DOI:** 10.1007/s10522-019-09849-8

**Published:** 2019-11-20

**Authors:** Dmytro V. Gospodaryov, Olha M. Strilbytska, Uliana V. Semaniuk, Natalia V. Perkhulyn, Bohdana M. Rovenko, Ihor S. Yurkevych, Ana G. Barata, Tobias P. Dick, Oleh V. Lushchak, Howard T. Jacobs

**Affiliations:** 1 Department of Biochemistry and Biotechnology, Vasyl Stefanyk Precarpathian National University, Ivano-Frankivsk, Ukraine; 2 Division of Redox Regulation, DKFZ-ZMBH Alliance, German Cancer Research Center (DKFZ), Heidelberg, Germany; 3 Faculty of Medicine and Health Technology, Tampere University, Tampere, Finland; 4 Present Address: Faculty of Biological and Environmental Sciences, and Institute of Biotechnology, University of Helsinki, Helsinki, Finland; 5 Department of Biochemistry and Biotechnology, Faculty of Natural Sciences, Vasyl Stefanyk Precarpathian National University, 57 Shevchenko Str, Ivano-Frankivsk, 76018 Ukraine

**Keywords:** Mitochondria, *Drosophila melanogaster*, Reactive oxygen species, Antioxidant defence, Stress resistance

## Abstract

Mitochondrial alternative NADH dehydrogenase (aNDH) was found to extend lifespan when expressed in the fruit fly. We have found that fruit flies expressing aNDH from *Ciona intestinalis* (NDX) had 17–71% lifespan prolongation on media with different protein-tocarbohydrate ratios except NDX-expressing males that had 19% shorter lifespan than controls on a high protein diet. NDX-expressing flies were more resistant to organic xenobiotics, 2,4-dichlorophenoxyacetic acid and alloxan, and inorganic toxicant potassium iodate, and partially to sodium molybdate treatments. On the other hand, NDX-expressing flies were more sensitive to catechol and sodium chromate. Enzymatic analysis showed that NDX-expressing males had higher glucose 6-phosphate dehydrogenase activity, whilst both sexes showed increased glutathione *S*-transferase activity.

**Electronic supplementary material:**

The online version of this article (10.1007/s10522-019-09849-8) contains supplementary material, which is available to authorized users.

## Introduction

Many organisms, but not insects, contain unconventional, ‘alternative’ enzymes in their mitochondrial respiratory chains (RC) (Matus-Ortega et al. 2011; McDonald and Gospodaryov 2019). These enzymes catalyse reactions similar to those catalysed by the conventional RC complexes, except that they do not pump protons across the inner mitochondrial membrane. Alternative NADH dehydrogenase (aNDH) or type II NADH dehydrogenase can bypass complex I of the conventional RC, whilst alternative ubiquinol oxidase (AOX) can provide a bypass of complexes III and/or IV. Functions of the aNDHs and AOXs in different organisms are not fully elucidated. It is believed that these enzymes provide larger flexibility of the respiratory chain, allowing adaptation to frequent changes in the levels of oxygen in the environment or transitions from anaerobic to aerobic conditions and vice versa (McDonald and Gospodaryov 2019). Alternative NADH dehydrogenase, similarly to complex I of the conventional RC, reduces ubiquinone, taking electrons from NADH, but does not pump protons across inner mitochondrial membrane. Unlike complex I that has multiple subunits encoded by both nuclear and mitochondrial DNA, aNDH comprises only a single gene product that can thus be easily cloned and expressed in a target cell. The ability of aNDH to bypass deficiencies in subunits of conventional RC complexes has been confirmed by many studies since early 2000s (Yagi et al. 2006; Perales-Clemente et al. 2008; Sanz et al. 2010; Santidrian et al. 2013). In parallel, it was found that transgenic animals which contain aNDH from other species demonstrated prolonged lifespan (Sanz et al. 2010; Bahadorani et al. 2010; Hur et al. 2013; Gospodaryov et al. 2014). Along with prolonged lifespan, aNDH provided resistance to such toxicants as paraquat and menadione (Sanz et al. 2010; Bahadorani et al. 2010; Gospodaryov et al. 2014), and to multiple stresses such as cold, heat and starvation (Gospodaryov et al. 2014).

In the majority of above-mentioned studies, lifespan extension was achieved by using Ndi1, an aNDH derived from the budding yeast *Saccharomyces cerevisiae*. In 2014, we first revealed that the enzyme of animal origin, from tunicate *Ciona intestinalis*, was able to prolong lifespan of the fruit fly *Drosophila melanogaster* being heterologously expressed in its body (Gospodaryov et al. 2014). Whatever the source of the enzyme, the mechanism of the lifespan extension by means of aNDH remained obscure. Alternative NDH allows a partial bypass of mitochondrial RC complex I, one of the main generators of reactive oxygen species (ROS) which damage essential cellular components, including mitochondrial DNA. Therefore, it was assumed and subsequently shown that isolated mitochondria with Ndi1 (aNDH from the budding yeast) released less hydrogen peroxide than those in the control (Sanz et al. 2010). Recently, it was found that tissues of Ndi1-expressing flies produced more ROS (Scialò et al. 2016). It was suggested that increased ROS production of Ndi1-expressing fruit flies is achieved by means of reverse electron transport from ubiquinol, a reduced form of RC electron carrier ubiquinone, to complex I. Following this line, ROS could extend lifespan only if they are involved into mitohormesis (Ristow 2014; Yun and Finkel 2014; Sanz 2016). However, the target of ROS in the organisms which express aNDH from whatever source was not yet found. Based on these previous findings, we have chosen following criteria to locate the biochemical pathway affected by excessive ROS in aNDH-expressing organisms: (a) it should be activated by ROS, (b) it should provide resistance of the organism to multiple stresses, (c) the lifespan extension should not substantially depend on the dietary macronutrient balance.

A link between stress resistance and long lifespan has recently been proposed and shown to be mediated by the transcription factors FOXO and Nrf2 (Sykiotis and Bohmann 2008; Murugaiyah and Mattson 2015; Castillo-Quan et al. 2016). The transcription factor Nrf2 was shown to regulate response of the organism to xenobiotics by boosting expression of enzymes involved in their detoxification. Activities of some of these enzymes are relatively easy measurable, e.g. glutathione *S*-transferase and glucose 6-phosphate dehydrogenase.

In a previous study (Gospodaryov et al. 2014), we have shown that aNDH from tunicate *Ciona intestinalis* (NDX), similarly to the yeast-derived enzyme Ndi1 (Sanz et al. 2010), extends lifespan in fruit fly model. In the current study, using transgenic fruit fly model, we demonstrate that a metazoan aNDH extends lifespan on diets with different macronutrient ratios with the smallest effect on high protein diets. We show that the lifespan extension occurs due to increase in rather age-independent mortality, i.e. decrease in frailty of young individuals. The enzyme also provided resistance of flies to toxic substances such as 2,4-dichlorophenoxyacetic acid, alloxan, potassium iodate, whereas it sensitized their organism to others, such as catechol and sodium chromate. We also show that aNDH-expressing flies have higher activity and expression of some Nrf2 targets, namely glutathione *S*-transferase and glucose 6-phosphate dehydrogenase.

## Methods

### Fruit fly lines and husbandry

Both control flies and the line expressing alternative rotenone-insensitive NADH dehydrogenase from *Ciona intestinalis* (NDX) were created on the background of the attP-bearing line *y*^*1*^w*; PBac{*y*^+^-attP-3B}VK00001 (Venken et al. 2006). Vector pUASTattB (GenBank accession number EF362409.1) was injected into embryos of the background line to obtain control flies, while transgenic vector pUASTattB-NDX was used for creating NDX-expressing flies (Gospodaryov et al. 2014; Figs. S1a and S1b). The injection of the vector into background lines was performed by Rainbow Transgenic Flies, Inc. (Camarillo, CA, USA). Transgenic control and NDX lines were further crossed with a line expressing GAL4 under the *daughterless* promoter (*da*-*GAL4*) (Wodarz et al. 1995) to obtain transgenic lines homozygous for the GAL4 driver on chromosome 3. The NDX-expressing line was also homozygous for NDX on chromosome 2. Targeting of NDX into mitochondria and its co-localization with mitochondrial proteins was demonstrated previously, as well as its expression in adult flies (Gospodaryov et al. 2014). In this study, NDX was expressed in adult flies (genotypes are depicted on Fig. S1c) without any tag. The expression was also confirmed by Western Blot (Gospodaryov et al. 2014; Fig. S1d). Possible predicted sites of mitochondrial targeting sequence are represented in Table S1.

Lines were cultured in 250 ml glass bottles at a density of approximately 200–300 eggs per bottle on medium containing 5% dry yeast, 6% fine corn groats, 7.5% molasses, 0.18% 4-hydroxybenzoic acid methylester (nipagin), and 0.4% propionic acid. The cultures were maintained at 25 ± 1 °C, 60% humidity, and 12:12 h light:dark cycle. For toxicity and lifespan assays, newly eclosed flies were separated by sex and either collected into 250 ml glass bottles or placed into glass vials with fresh medium. The assays were conducted 48 h after collection of eclosed flies into vials and bottles. Majority of females used in experiments were virgin since they were collected soon after eclosion.

### Lifespan analysis

Fruit fly cohorts of 200–300 flies (males and virgin females kept separately) were maintained in 1.5 l mortality cages (Gospodaryov et al. 2014). About 100–150 individuals were placed in one cage. Data were pooled from two independent experiments. The food contained baker’s yeast (Y) and sucrose (S) in different concentrations along with 1.2% agar, and 0.18% nipagin. Dead individuals were recorded every second day and removed from the cage.

### Toxicity assays

All experiments were conducted on 2–3 day-old flies. Sodium chromate and molybdate, potassium iodate, alloxan, and 2,4-diphenoxyacetic acid (2,4-D) were added into solidified medium containing 5% sucrose, 5% yeast, 1.5% agar and 0.18% nipagin. Concentrations of each substance are provided below in the text. Ten flies were maintained in each test vial with a toxicant until death. Every other day, flies were placed into new vials with fresh medium containing toxicant. Survivors were counted every 12 h.

Because of low stability and sensitivity to temperature, catechol was diluted in 5% sucrose solution and the resulting solutions were used to soak strips (2.4 cm × 12 cm) of four-layer cellulose filter paper compressed into the bottom of 1.5 cm × 15 cm glass test tubes. Ten flies were placed into each test tube and maintained until death. Survivors were counted every 12 h. Each assay was repeated five times. Therefore, fifty flies of certain genotype and sex groups were tested for each concentration of toxicant. Experiments with chromium and molybdate salts were conducted for three age groups, 7-, 14-, and 28-day-old flies.

### Enzymatic assays

Fruit fly homogenates were prepared as described by Lozinsky et al. (2013). Briefly: for all enzymes except superoxide dismutase (SOD), 10–15 flies were homogenized in a glass-on-glass tapered tissue grinder (1:10 w/v) in cold 50 mM potassium phosphate (KPi) buffer (pH 7.5), containing 0.5 mM *N*,*N*,*N*′,*N*′-ethylenediaminetetraacetic acid (EDTA) and 1 mM phenylmethylsulfonyl fluoride. After centrifugation at 16,100×*g* for 15 min at 4 °C, the supernatants were collected and used for enzymatic assays and estimation of protein concentration. Activities of SOD, glucose 6-phosphate dehydrogenase (G6PDH), NADP^+^-specific malate (MDH) and isocitrate dehydrogenases (ICDH) were measured, using assays described by Lushchak et al. (2005); glutathione *S*-transferase (GST) and lactate dehydrogenase (LDH) were measured as described by Bagnyukova et al. (2005) and Kubrak et al. (2013), respectively. In brief: the activity of SOD was assessed spectrophotometrically at wavelength 406 nm by its ability to inhibit oxidation of quercetin by superoxide anion-radical. About 40–50 flies were used for the measurement. The reaction mixture contained (final concentrations): 30 mM Tris–HCl buffer (pH 10.0), 0.5 mM EDTA, 0.8 mM *N*,*N*,*N*′,*N*′-tetramethylethylenediamine, 0.05 mM quercetin, and 2–100 μl of the supernatant. The reaction was measured for 6–8 different volumes of the supernatant. One unit of SOD activity was defined as the amount of enzyme (per milligram protein) that inhibits quercetin oxidation by 50% of maximum. Activities of G6PDH, MDH, ICDH, and LDH were measured spectrophotometrically at 340 nm by the rate of NAD(P)H formation/oxidation. The reaction mixture for G6PDH contained 50 mM KPi buffer (pH 7.5), 0.5 mM EDTA, 5 mM MgCl_2_, 0.2 mM NADP^+^, and 2 mM glucose 6-phosphate; for MDH—50 mM KPi buffer (pH 7.5), 7.5 mM MgCl_2_, 0.15 mM NADP^+^, and 1 mM l-malic acid; for ICDH—50 mM KPi buffer (pH 7.5), 2 mM MgCl_2_, 1 mM NADP^+^, 0.5 mM isocitric acid; for LDH—50 mM KPi buffer (pH 7.5), 0.5 mM EDTA, 1 mM pyruvate and 0.2 mM NADH. In all cases, reaction was started by adding 20 μl of the supernatant to 980 μl of the reaction mixture. The extinction coefficient 6.22 mM^−1^ cm^−1^ for NAD(P)H was used. One unit of G6PDH, ICDH, MDH, GST, and LDH activity was defined as the amount of the enzyme consuming 1 μmol of substrate or generating 1 μmol of product per minute; activities were expressed as international units (or milliunits) per milligram protein. The activity of GST was monitored at 340 nm by the formation of an adduct between reduced glutathione (GSH) and 1-chloro-2,4-dinitrobenzene (CDNB). The reaction mixture contained 50 mM KPi buffer (pH 7.5), 0.5 mM EDTA, 5 mM GSH, 1 mM CDNB, and 1–5 μl of the supernatant in a final volume of 1 ml. The reaction was initiated by the sequential addition of CDNB and supernatant. Blanks contained no CDNB. The extinction coefficient 9.6 mM^−1^ cm^−1^ for 1-*S*-glutathionyl-2,4-dinitrobenzene was used for calculation of the activity.

The protein concentration was measured by the Bradford method with Coomassie Brilliant Blue G-250 and using bovine serum albumin as a standard.

### Quantitative real-time polymerase chain reaction (QRTPCR)

Thirty male or twenty female flies from each of five independent repeats were collected and submerged in 1.4 ml of RNAlater reagent (Thermo Fisher Scientific). Total RNA was isolated using the RNeasy kit (QIAGEN Inc.) in accordance with the manufacturer’s protocol. mRNA was reverse transcribed using the RevertAid Premium reverse transcriptase (Thermo Fisher Scientific). Briefly, 5 mM of oligo(dT), 1 mM of deoxyribonucleotide triphosphates and 5 μg of total RNA were mixed in a PCR tube to a total volume of 10 μl, incubated for 5 min at 65 °C and stored on ice. In a second tube, 200 units (U) of RevertAid Premium reverse transcriptase were mixed with 1 × reverse transcriptase buffer and 40 U of RiboLock RNase inhibitor (Thermo Fisher Scientific) in a total volume of 10 μl. Finally, the volume in the two tubes was combined and incubated in a thermocycler for 5 min at 50 °C, 5 min at 85 °C, 1 s at 22 °C and stored on ice. The 2 × Maxima SYBR Green/ROX qPCR Master Mix (Thermo Fisher Scientific) was mixed with extra ROX Reference dye to a final concentration of 0.5 μM. Per well of a 96-well plate, the cDNA samples to be analysed were diluted 1:75 or 1:150 and mixed with 1 × SYBR Green/ROX Reference Dye and 0.25 μM of the forward and reverse primers.

### Statistical analysis

Lifespan curves were compared pairwise, using Mantel–Haenszel (log-rank) test (Harrington 2005). The difference between curves was considered significant at *p* value < 0.05. The calculations were performed in R (package *survival*). Parameters of age-dependent and age-independent mortalities were calculated by the modified Gompertz equation (Lushchak et al. 2012). The fitting of survival curves was performed using R package *minpack.lm*. Standard errors in Fig. 2 were calculated for the estimates of the regression model (*n* = 35–53), basing on average distances from the observed data points to the regression curve. Dietary response surfaces were fitted and drawn using R packages *mgcv* and *fields* (Semaniuk et al. 2018). Data of toxicity and enzymatic assays were compared, using Welch’s modification of two-tailed Student’s *t* test (*p* < 0.05) and represented as mean ± standard error of the mean. Median lifespan in toxicity assays was calculated as time at which 50% of a cohort died. Calculations were performed by means of Microsoft Excel.

## Results

### NDX prolongs lifespan on media with different protein-to-carbohydrate ratios

Activations of FOXO (***fo****rkhead* bo**x O**) and AMPK (**a**denosine **m**onophosphate-activated **p**rotein **k**inase) signalling pathways have been shown to extend lifespan (Hay 2011). This extension can be regulated by diet and both signalling pathways are thought to be targets for dietary restriction and its mimetics (Lushchak and Gospodaryov 2017). However, some anti-aging drugs or mutations in genes which activate these signalling pathways do not work on calorically restricted diets (Giannakou et al. 2008; Tatar et al. 2014). Therefore, to learn whether lifespan extension provided by aNDH depends on a dietary macronutrient balance, we tested a broader range of diets than it was used in earlier studies (Sanz et al. 2010; Gospodaryov et al. 2014).

**Fig. 1:**
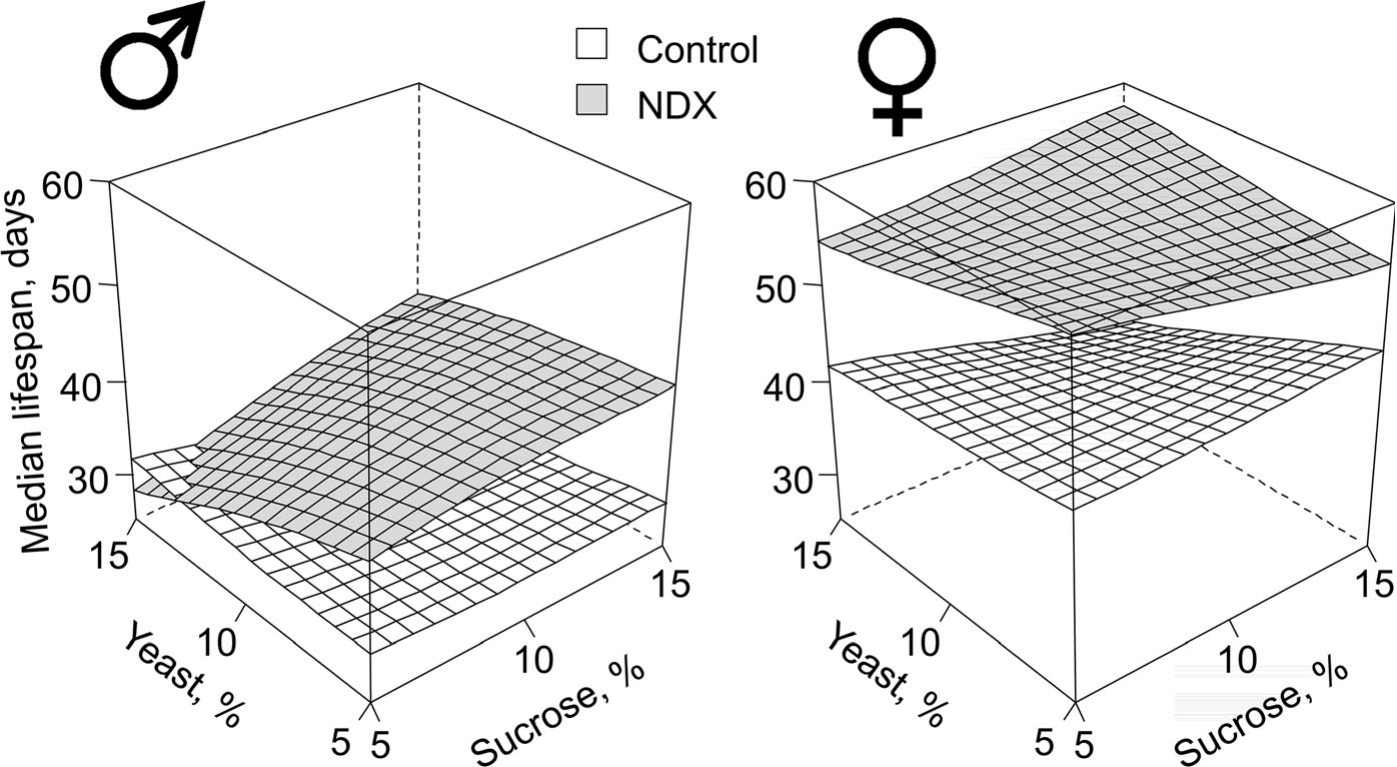
Response surfaces showing dependency of median lifespans in control and NDX-expressing flies on dietary carbohydrate and yeast. Standard symbols denote male and female flies, respectively. Differences between lifespans on all diets are significant. Exact *p* values for comparisons using Mantel–Haenszel test are represented in Tables 1 and 2. From 221 to 302 individuals were used per line and diet

The NDX-expressing flies had from 17 to 71% longer median lifespan on media with different combinations of yeast and sucrose (Figs. 1, S2; Tables 1, 2). In other words, lifespan extension provided by NDX was present regardless of diet composition but varied in degree on different diets. An exception was observed only for NDX-expressing males kept on the medium 5S:15Y (5% sucrose and 15% yeast), on which median lifespan was decreased by 19% compared to control males. Among NDX-expressing males, the biggest lifespan extensions, about 50% increase, compared to corresponding controls, were observed for those kept on media 10S:10Y and 15S:15Y. Among NDX-expressing females, the smallest lifespan extension, about 17% of median lifespan from the control, was observed on 15S:5Y diet, while the biggest extension was on the 15S:15Y diet.

**Table 1:** *p* Values for comparison of lifespan curves of the control and NDX-expressing male flies on different diets using Mantel–Haenszel (log-rank) test (n = 221–299)

Males						
	Diet	5S:5Y	5S:15Y	10S:10Y	15S:5Y	15S:15Y
Median lifespan	Control	30	32	28	30	26
	NDX	40	26	42	40	38
	p-value	1.26 × 10^−7^	3.70 × 10^−2^	< 1.00 × 10^−16^	1.00 × 10^−8^	6.66 × 10^−16^
	% change	33.3	− 18.8	50.0	33.3	46.2

**Table 2:** *p* Values for comparison of lifespan curves of the control and NDX-expressing female flies on different diets using Mantel–Haenszel (log-rank) test (n = 262–302)

Females						
	Diet	5S:5Y	5S:15Y	10S:10Y	15S:5Y	15S:15Y
Median lifespan	Control	44	42	41	46	34
	NDX	60	54	56	54	58
	p-value	< 1.00 × 10^−16^	< 1.00 × 10^−16^	< 1.00 × 10^−16^	3.85 × 10^−5^	< 1.00 × 10^−16^
	% change	36.4	28.6	36.6	17.4	70.6

**Fig. 2:**
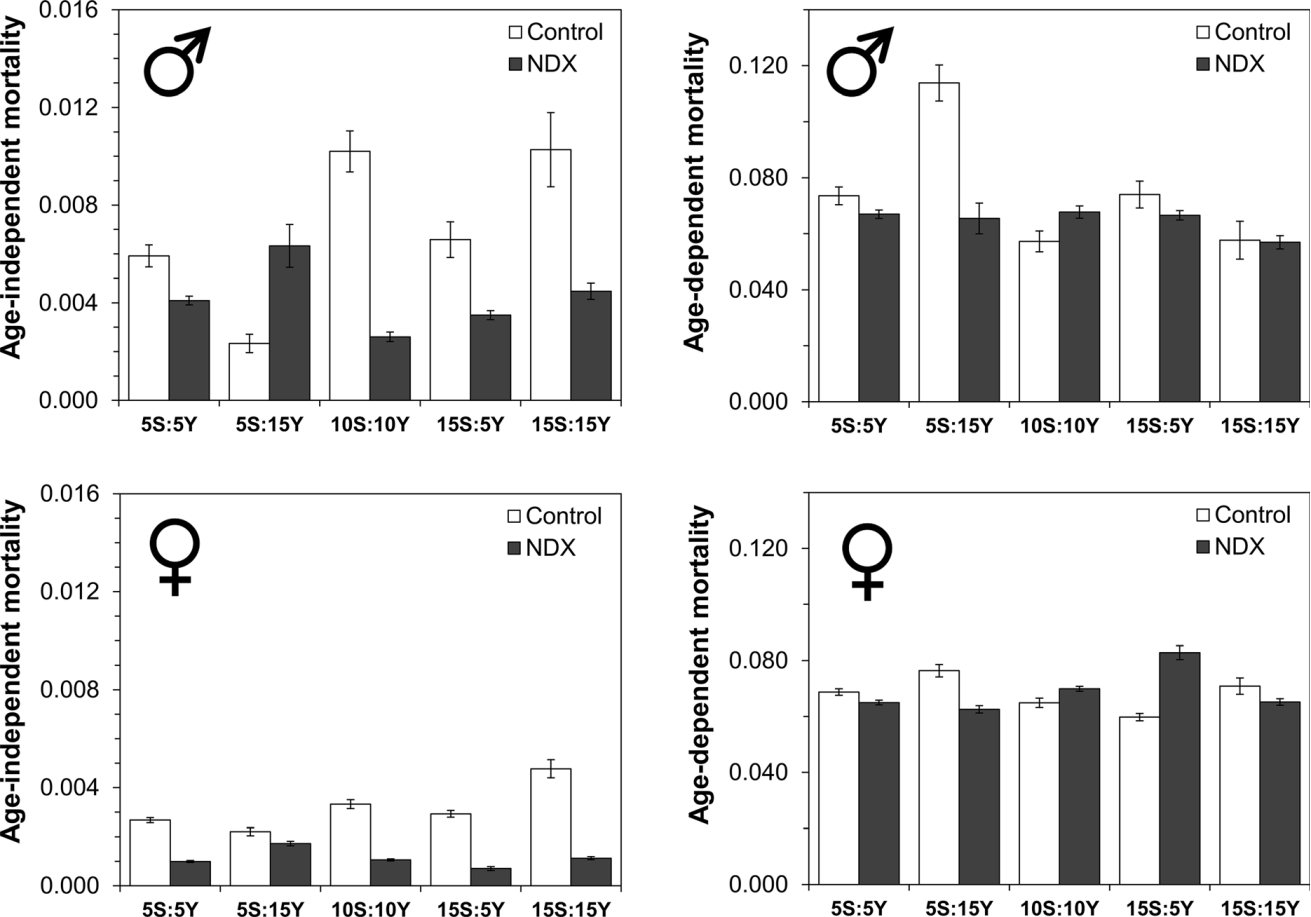
Estimates of age-dependent and age-independent mortalities calculated by modified Gompertz equation (Lushchak et al. 2012). Standard symbols denote male and female flies, respectively. Data are presented as estimates ± standard errors (n = 35–53)

Interestingly, the observed lifespan extension in NDX-expressing flies was due to drastic, 1.4–4.2-fold, decrease in age-independent mortality (Figs. 2, S3, S4). On the other hand, age-dependent mortality was not affected substantially. This suggests that NDX increases robustness of frail individuals within the cohort, thus decreasing mortality of young individuals.

### NDX provides resistance to xenobiotics and inorganic toxicants

Our data suggest that lifespan extension in fruit flies expressing aNDH is likely mediated by a cellular signalling pathway which is not strongly diet-dependent. A weak dependence of NDX-induced lifespan extension on diet invites consideration of alternative regulatory pathways. One of the candidates is the Nrf2 pathway, which is activated in long-living rodents, such as naked mole rats (Bruns et al. 2015), and under lithium-induced lifespan extension in *D. melanogaster* (Castillo-Quan et al. 2016). Nrf2 is known to be responsible for regulation of the response to xenobiotics (Misra et al. 2011; Blackwell et al. 2015; Holmström et al. 2016). Therefore, we tested NDX-expressing flies for susceptibility to xenobiotics.

**Fig. 3:**
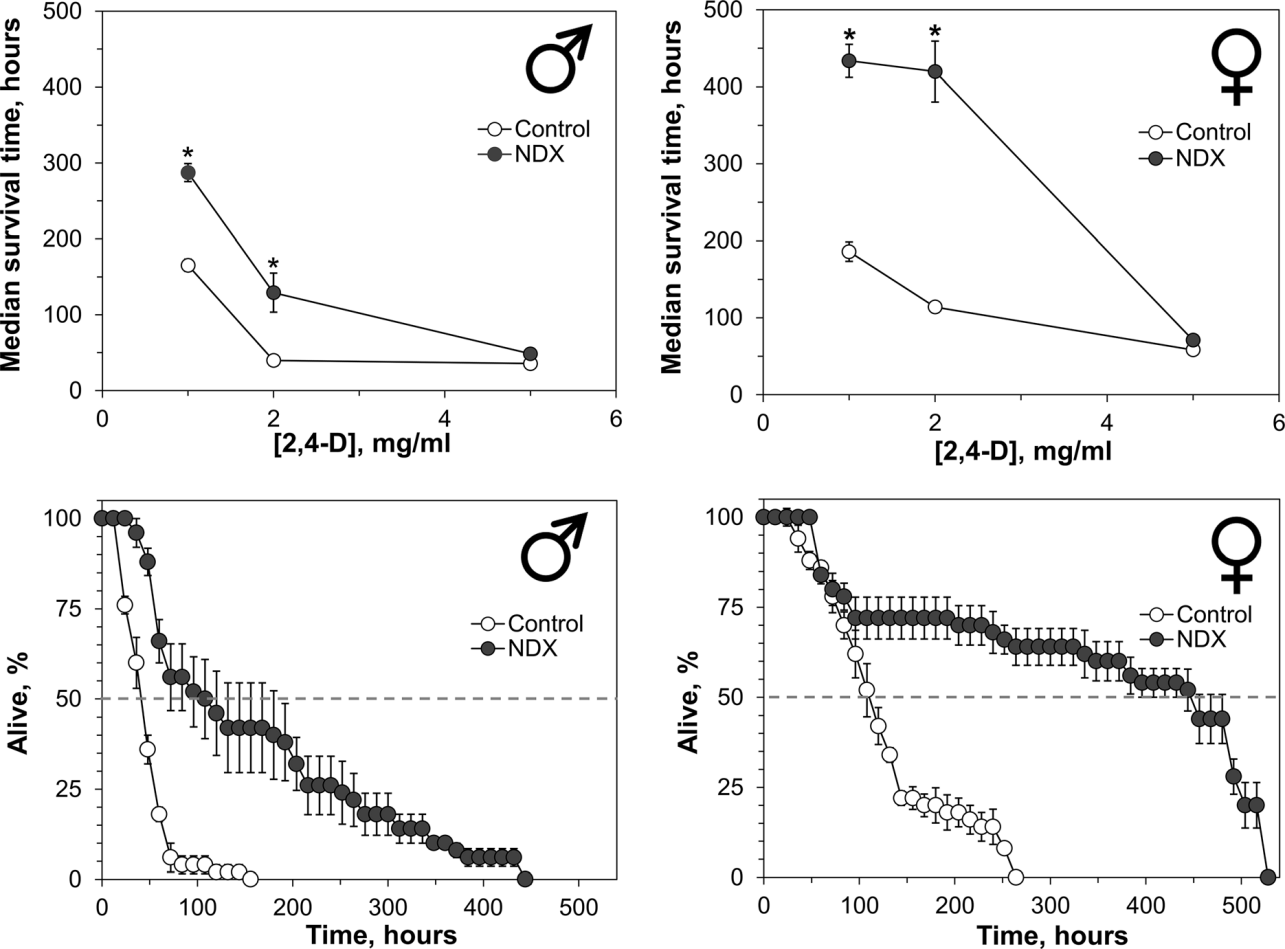
Resistance of NDX-expressing flies to different concentrations 2,4-dichlorophenoxyacetic acid (2,4-D; upper panel) and survival curves for cohorts kept on 2 mg ml^−1^ 2,4-D (lower panel). Standard symbols denote male and female flies. Each point of the curves represents mean ± SEM. Asterisks on the plots in upper panel show significant difference between median survival times compared by two-tailed Welch’s *t* test (*p* < 0.05, n = 5)

**Fig. 4:**
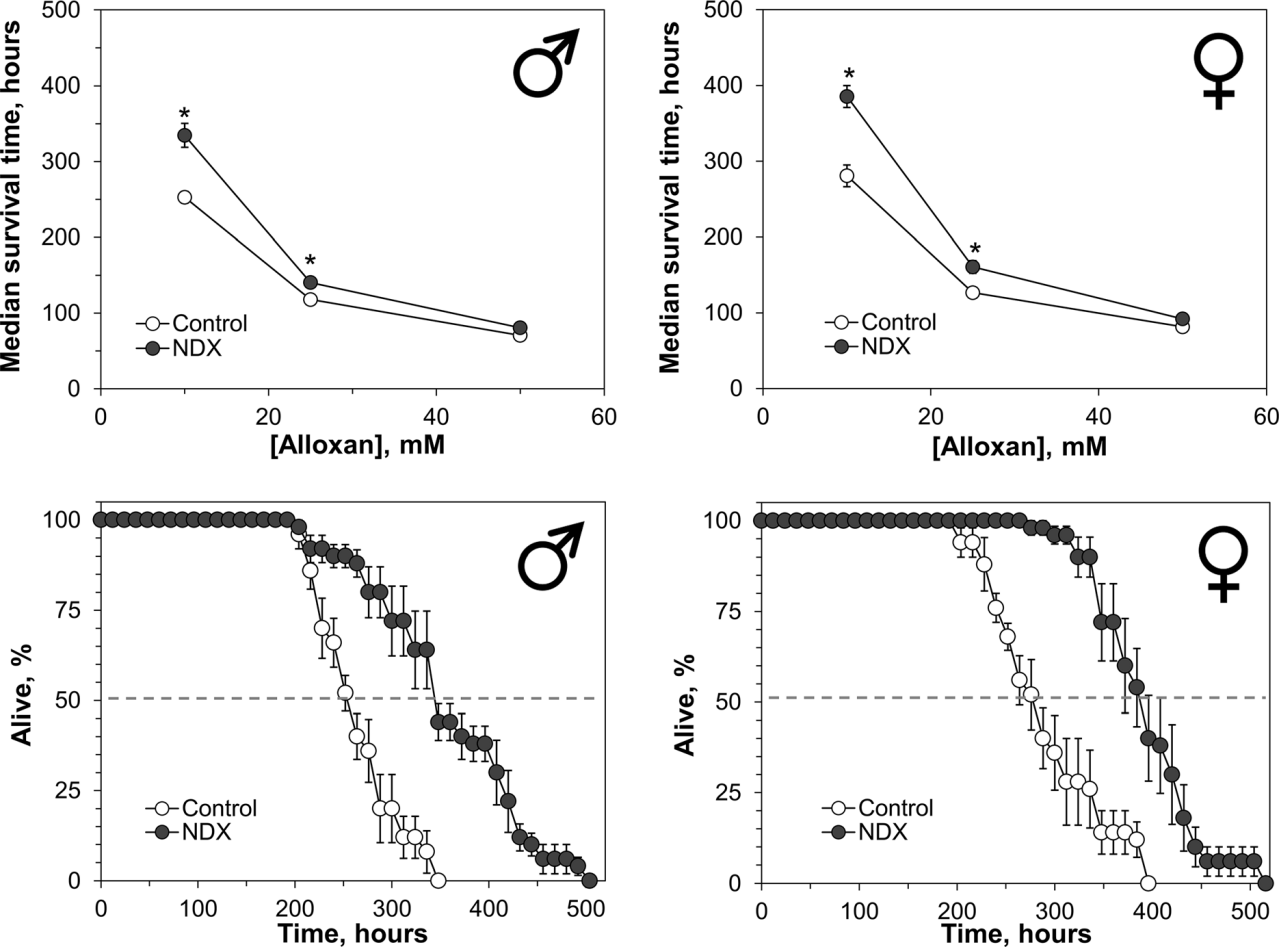
Resistance of control and NDX-expressing flies to different concentrations of alloxan (upper panel) and survival curves for cohorts on kept 10 mM alloxan (lower panel). Standard symbols denote male and female flies. Each point of the curves represents mean ± SEM. Asterisks on the plots in upper panel show significant difference between median survival times compared by Welch’s t-test (*p* < 0.05, n = 5)

**Fig. 5:**
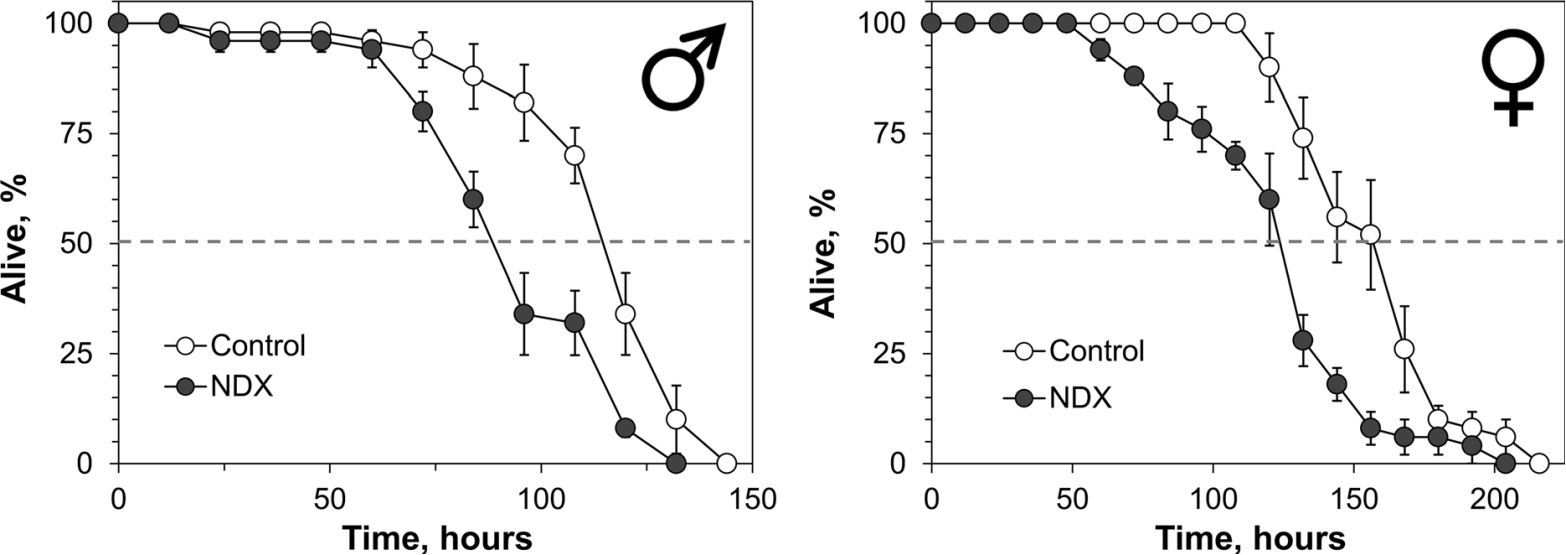
Resistance of control and NDX-expressing flies to 100 mM catechol. Standard symbols denote male and female flies. Each point of the curves represents mean ± SEM from five independent repeats of the experiment. Asterisks on the plots in upper panel show significant difference between median survival times compared by Welch’s *t* test (*p* < 0.05, n = 5)

Among organic toxicants, we used 2,4-dichlorophenoxyacetic acid (2,4-D), alloxan, and catechol, some of which were formerly studied in our laboratory on different models (Abrat et al. 2005; Atamaniuk et al. 2013). We also tested several inorganic compounds such as potassium iodate, sodium molybdate and sodium chromate as stressors for NDX-expressing flies. The latter two salts were also tested previously in *Drosophila* (Rovenko et al. 2014; Perkhulyn et al. 2015, 2017). It is well documented that exposure to 2,4-D or alloxan results in depletion of reduced glutathione levels in tissues (Lenzen 2008; Atamaniuk et al. 2013). In turn, Nrf2 was shown to be activated by depletion of reduced glutathione levels (Chia et al. 2010). Catechol is known as Nrf2 activator (Wang et al. 2010), therefore we used it as the control in addition to 2,4-D and alloxan, less studied in insect models. The largest difference between median lifespans of the investigated lines was observed at 2 mg ml^−1^ of 2,4-D (Fig. 3). At this concentration, flies which expressed NDX had three to fourfold longer median lifespan than the control individuals. A higher concentration of 2,4-D abrogated the difference. Similar dependence on concentration was found for alloxan, a redox-cycling compound (Fig. 4). The largest difference (24% and 27% longer median lifespan of NDX-expressing male and female flies, respectively) between the lines was observed at 10 mM of the toxicant. At a 2.5-fold higher concentration of alloxan, the difference was still significant but much smaller, while a fivefold higher concentration abrogated the difference. Catechol at 100 mM shortened median lifespan of NDX-expressing male and female flies by 19% and 23% as compared with the control ones, respectively (Fig. 5).

**Fig. 6:**
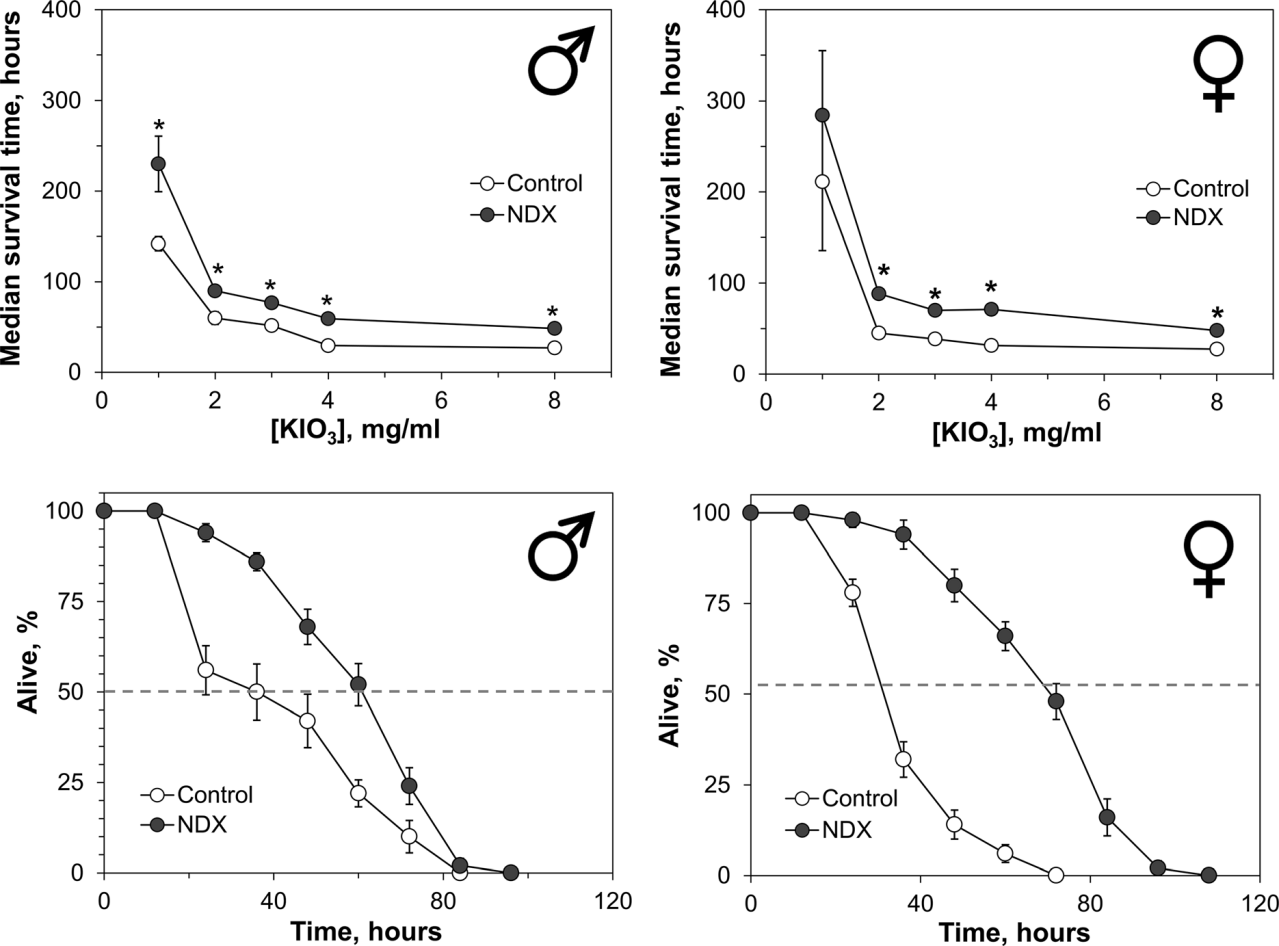
Resistance of control and NDX-expressing flies to different concentrations of potassium iodate (upper panel) and survival curves for cohorts kept on 4 mg ml^−1^ potassium iodate (lower panel). Standard symbols denote male and female flies. Each point of the curves represents mean ± SEM. Asterisks on the plots in upper panel show significant difference between median survival times compared by two-tailed Welch’s *t* test (*p* < 0.05, n = 5)

Flies, which express NDX, had 1.4–2.2-fold longer lifespan than controls, when kept on the medium with a strong oxidizer potassium iodate (Fig. 6). A steep drop in resistance to iodate was observed when its concentration was increased from 1 to 2 mg ml^−1^. Further increase in iodate concentration did not change resistance considerably and the NDX-expressing flies retained 1.5–2.0-fold higher resistance than controls.

**Fig. 7:**
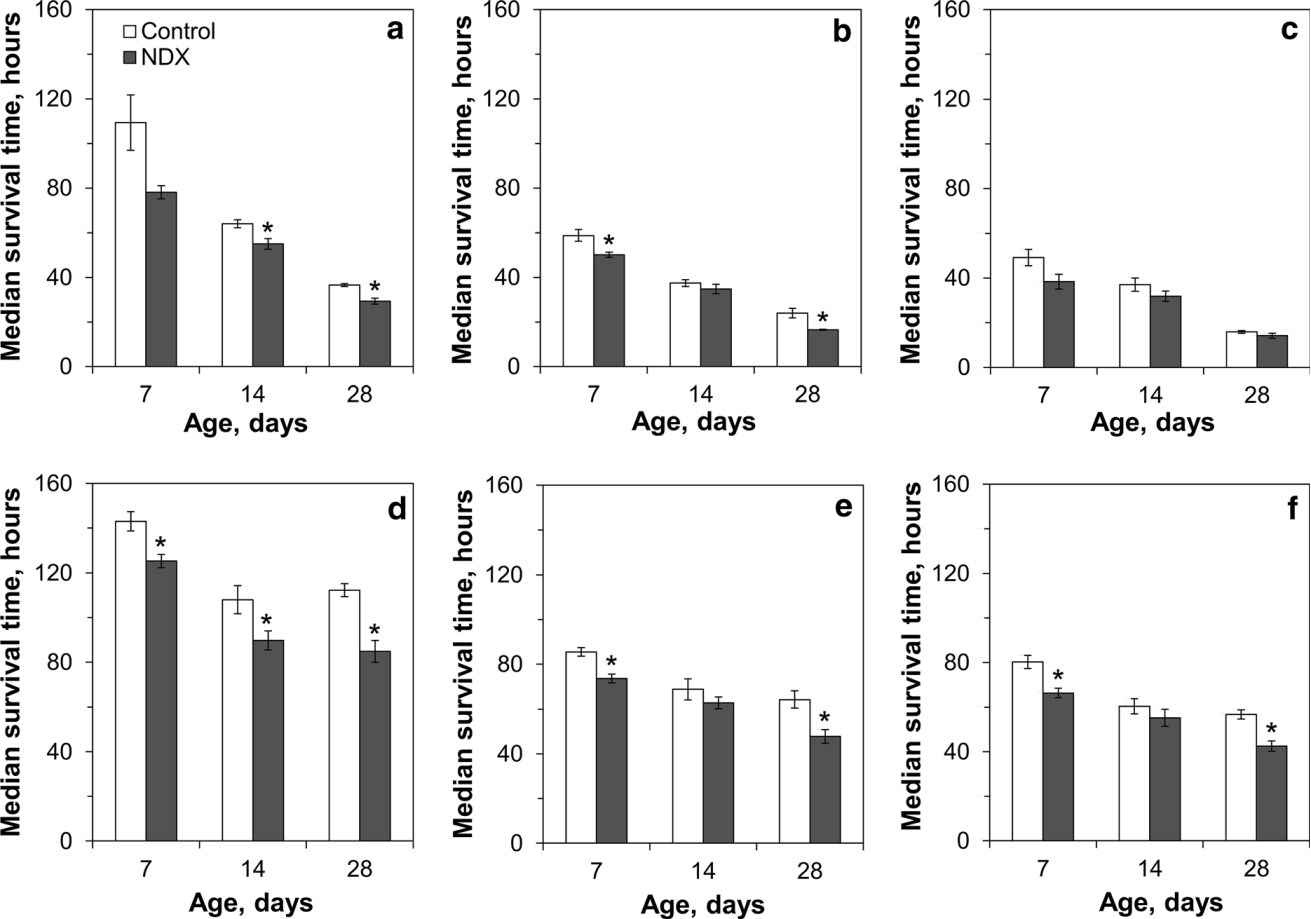
Resistance of control and NDX-expressing flies to different concentrations of sodium chromate: **a**, **d** 5 mM; **b**, **e** 25 mM; **c**, **f** 50 mM. **a**–**c** Median survival time for males, **d**–**f** median survival time for females. Asterisks show significant difference between median survival times compared by Welch’s *t* test (*p* < 0.05, n = 5)

**Fig. 8:**
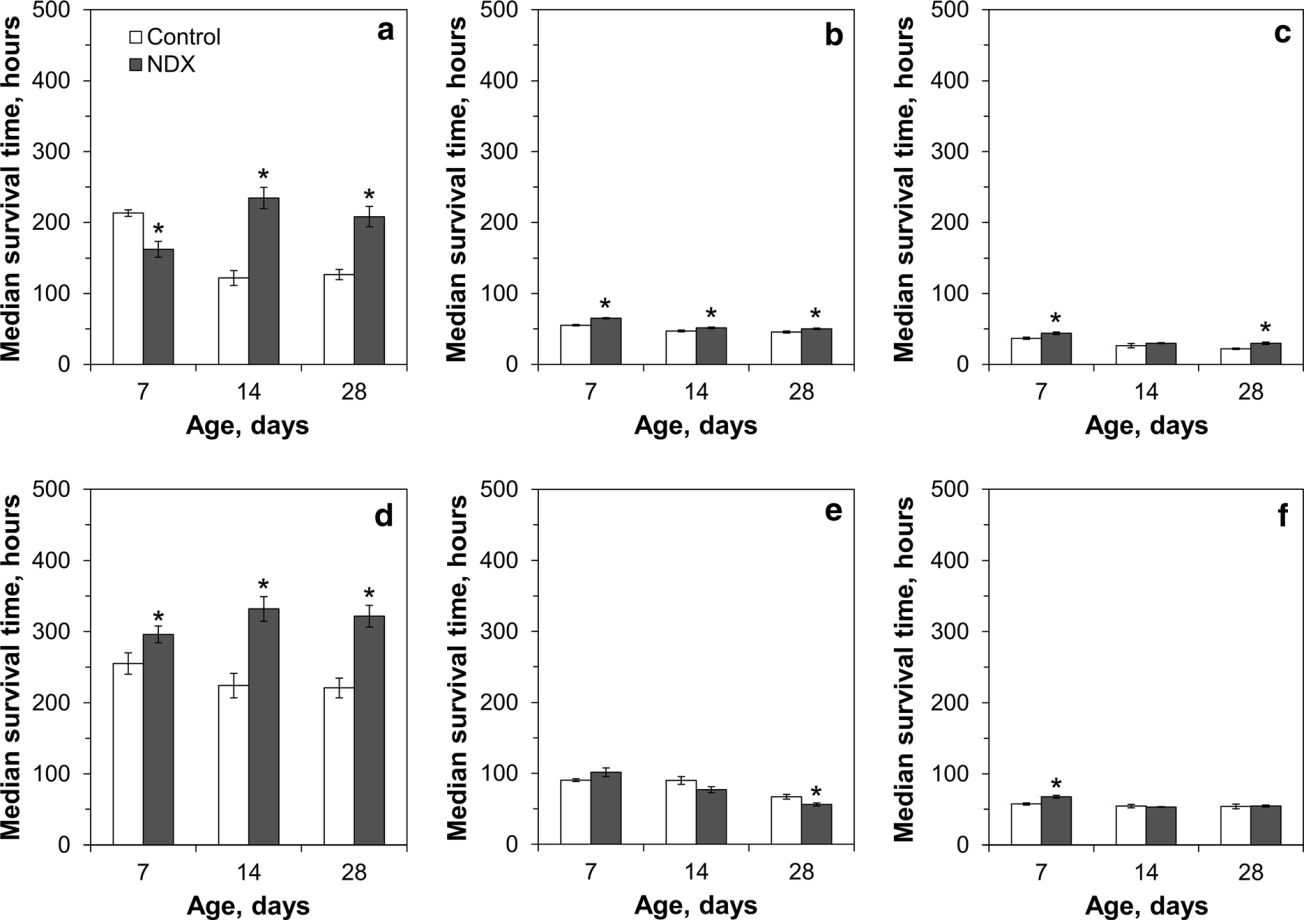
Resistance of control and NDX-expressing flies to different concentrations of sodium molybdate: **a**, **d** 100 mM; **b**, **e** 250 mM; **c**, **f** 500 mM. **a**–**c** Median survival time for males, **d**–**f** median survival time for females. Asterisks show significant difference between median survival times compared by Welch’s *t* test (*p* < 0.05, n = 5)

In all cases, chromate was more toxic for NDX-expressing flies than for control flies, but the strength of the effect was dependent on age, sex, and concentration (Table S2). The largest difference between 7-day-old control and NDX-expressing males was observed at 5 mM of chromate (Fig. 7; Table S2). The difference among 7-day-old control and NDX-expressing females fell in the range of 12–17% for all investigated concentrations of chromate. The NDX-expressing flies were more resistant to sodium molybdate than the control flies, although not in all cases (Fig. 8; Table S3). Particularly, NDX-expressing male flies had a 24% shorter median lifespan than the control when fed medium with 100 mM sodium molybdate (Fig. 8a). At the same time, NDX-expressing females were 14% more resistant to this concentration of molybdate, compared to control flies (Fig. 8d). At higher concentrations of molybdate, 250 and 500 mM, NDX-expressing flies had about 11–16% longer median lifespan than the control flies.

We checked whether the influence of inorganic toxicants would depend on the age of the flies. At most concentrations, sodium chromate conferred smaller differences between control and NDX-expressing flies at 14 than 7 days of age. However, 28-day-old NDX-expressing flies were again about 30% more sensitive to chromate than the control flies. A trend for increasing difference between control and NDX-expressing flies with age was observed for molybdate treatment, although there was no regular pattern or clear correlation. In particular, median lifespan of 14-day-old and 28-day-old NDX-expressing flies fed on food containing 100 mM sodium molybdate was 31–48% longer than in corresponding controls. At the same time, there was no difference in sensitivity between control and NDX-expressing female flies to 500 mM molybdate.

### NDX activates glutathione *S*-transferase (GST) and glucose 6-phosphate dehydrogenase (G6PDH)

Our current data on sensitivity to toxicants, together with the previous data on resistance of NDX-expressing flies to stresses and their lifespan extension, suggest that transcription factor Nrf2 might mediate those effects (Bruns et al. 2015). Nrf2 regulates the expression of tens of proteins, including different isoenzymes of cytochrome P450, involved in xenobiotic detoxification, enzymes responsible for glutathione synthesis, different isoenzymes of GST, NADP-reducing enzymes, such as G6PDH, NADP-dependent isocitrate dehydrogenase, malic enzymes, NAD(P)H:quinone reductase, heme oxygenase, and many others (Holmström et al. 2016). To investigate the involvement of Nrf2 in the increased resistance of NDX-expressing flies to toxicants, we checked the activity of enzymes whose expression has been shown to be regulated by Nrf2. For this study, we selected enzymes, whose expression was shown previously to rise in response to xenobiotics or mutations leading to activation of Nrf2 signalling (e.g. deletion or inactivation of Keap1, a negative regulator of Nrf2) (Misra et al. 2011, 2013; Castillo-Quan et al. 2016).

**Fig. 9:**
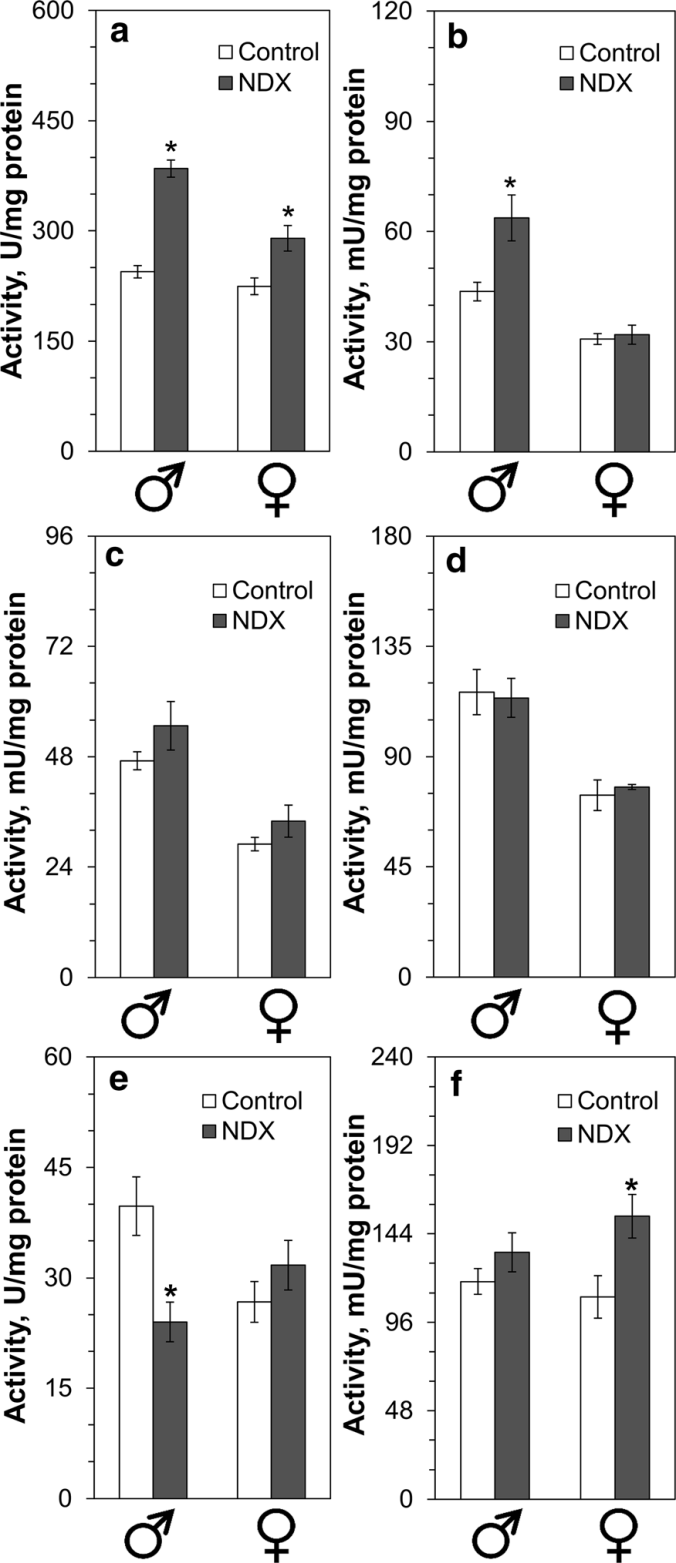
Activities of enzymes which are targets of transcription factor Nrf2 (encoded by the gene *CncC* in *Drosophila melanogaster*). Standard symbols denote male and female flies, respectively. Asterisks show significant difference (two-tailed Welch’s *t* test, *P* < 0.05, n = 4–5). Activities of **a** glutathione *S*-transferase, **b** glucose-6-phosphate dehydrogenase, **c** NADP-linked malate dehydrogenase, **d** NADP-linked isocitrate dehydrogenase, **e** SOD—superoxide dismutase, and **f** lactate dehydrogenase are shown

Activity of GST in NDX-expressing male and female flies was 1.6- and 1.3-fold higher than in corresponding control flies, respectively (Fig. 9a). Activity of G6PDH was 1.5-fold higher in NDX-expressing males as compared with controls, while no difference was observed in females (Fig. 9b). On the other hand, superoxide dismutase activity of NDX-expressing males was 1.5-fold lower as compared with controls and no significant difference was found in females (Fig. 9e). There was no difference between activities of NADP-reducing isocitrate and malate dehydrogenases in control and NDX-expressing flies, although the activity of both enzymes was higher in males (Fig. 9c, d).

Upregulation of the ecdysone-induced lactate dehydrogenase (LDH) has recently been shown in flies with RC defects (Fernández-Ayala et al. 2010). Surprisingly, NDX-expressing females had 1.4-fold higher LDH activity than corresponding controls (Fig. 9f). However, there was no difference in LDH activity between the control and NDX-expressing males.

### mRNA levels of Nrf2 targets

**Fig. 10:**
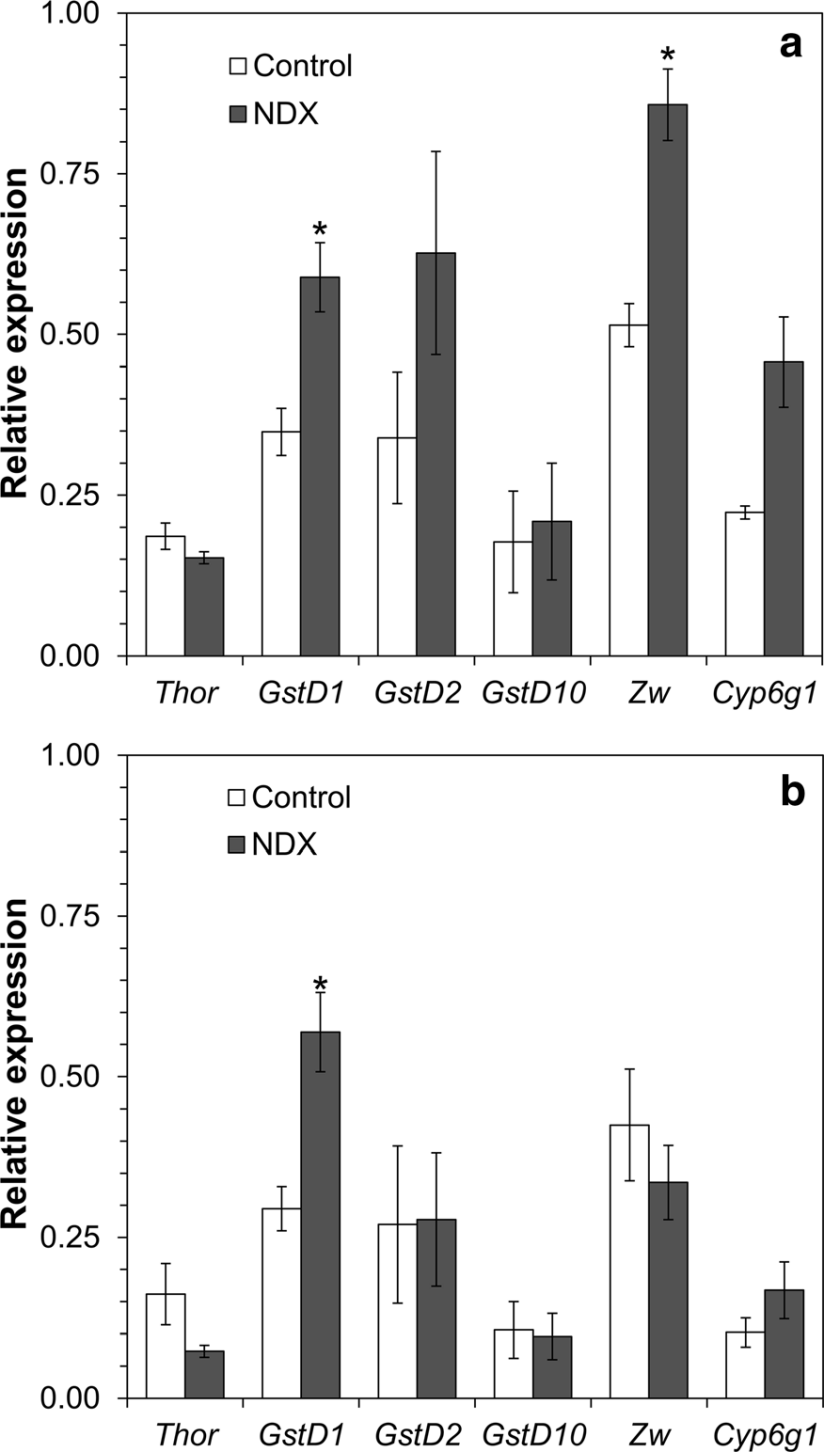
Transcript levels for specific *CncC* (*dNrf2*) targets in **a** males **b** females. Isoenzymes of glutathione *S*-transferase are *GstD1*, *GstD2*, *GstD10*, glucose 6-phosphate dehydrogenase—*Zw*, and cytochrome P450—*Cyp6g1*. All genes whose transcription was tested were chosen on the basis of previous data (Castillo-Quan et al. 2016) and enzymatic activity measurements in the current study. Asterisk denotes significant difference between control and NDX-expressing flies (two-tailed Welch’s *t* test, *p* < 0.05, n = 3–4). Due to differences in expression of housekeeping genes, transcript levels of studied genes were normalized against *RpL32* (for *Thor* and *GstD1*) or *nuf* transcript levels (*GstD2*, *GstD10*, *Zw*, *Cyp6g1*)

To investigate whether expression of chosen Nrf2 targets is regulated at the transcription level we assessed expression of *GstD1*, *GstD2*, and *GstD10*, which code for different isoenzymes of GST, and expression of *Zw* gene coding for G6PDH, *Cyp6g1* coding for a cytochrome P450, and *Thor* coding for eukaryotic translation initiation factor 4E-binding protein 1 (4EBP1), regulated by TOR kinase. *D. melanogaster* flies contain multiple isoenzymes of GST and cytochrome P450, whose expression was technically difficult for us to measure. Therefore we focused on the listed above isoenzymes, basing on the expression analysis published previously (Castillo-Quan et al. 2016). The latter allowed us to assess the possible involvement of the TOR pathway, well studied in regard to aging (Bitto et al. 2015). Transcript levels of *GstD1* were 1.7–1.9-fold higher in NDX-expressing flies of both sexes (Fig. 10). Transcripts of *Zw* were 1.7-fold elevated in NDX-expressing males but not females. These data are consistent with the differences in enzymatic activity of G6PDH between control and NDX-expressing flies.

## Discussion

### Lifespan extension in NDX-expressing flies

Prolonged lifespan in *D. melanogaster* flies expressing aNDH was shown in several independent studies (Sanz et al. 2010; Bahadorani et al. 2010; Gospodaryov et al. 2014). In the majority of cases, aNDH from the budding yeast *Saccharomyces cerevisiae* has been used. Initially, prolonged lifespan was explained in terms of the mitochondrial free radical theory of ageing (Sanz et al. 2010). Alternative NADH dehydrogenases can by-pass complex I of mitochondrial RC, which is considered to be one of the major generators of reactive oxygen species (ROS). However, it was recently shown that mitochondria of Ndi1p-expressing fruit flies actually exhibit a higher steady-state level of ROS than flies with just the conventional RC (Scialò et al. 2016). Therefore, the prolonged lifespan of these flies can be explained in terms of mitohormesis, a recently proposed but increasingly supported concept (Yang and Hekimi 2010; Yun and Finkel 2014; Ristow 2014; Sanz 2016). It means that ROS produced by mitochondria activate pro-survival and pro-longevity processes and pathways in cells. Several signalling pathways have been shown to be strongly associated with ageing, notably the insulin, target-of-rapamycin (TOR) and adenosine monophosphate (AMP)-activated protein kinase (AMPK) pathways (Bitto et al. 2015). Cross-talk between these pathways has also been demonstrated (Burkewitz et al. 2014). Recently, the signalling pathway governed by the transcriptional regulator Nrf2 (nuclear factor (erythroid-derived 2)-like 2) was found to have influence on ageing (Sykiotis and Bohmann 2008; Burkewitz et al. 2014; Blackwell et al. 2015; Bruns et al. 2015; Castillo-Quan et al. 2016; Lushchak and Gospodaryov 2017). There are several pieces of evidence implicating Nrf2 in regulating cell senescence along with the insulin, TOR, and AMPK pathways. The Nrf2 pathway is a strong candidate to account for mitohormesis, since this pathway is triggered by ROS (Blackwell et al. 2015). The relative expression of Nrf2-regulated genes, specifically NAD(P)H:quinone reductase, GST, and heme oxygenase, is elevated in the naked mole rat (*Heterocephalus glaber*), a long-lived rodent, compared with mice (Lewis et al. 2015). Nrf2 DNA-binding capacity also correlates with lifespan in different rodent species (Lewis et al. 2015). Considerable evidence supporting a key role for Nrf2 in the regulation of lifespan in *D. melanogaster* has been reported (Sykiotis and Bohmann 2008; Pitoniak and Bohmann 2015). It was recently found that the pro-longevity effects of lithium preparations in *Drosophila* model were associated with Nrf2 activation (Castillo-Quan et al. 2016). Involvement of Nrf2 in lifespan extension is not limited to rodents or fruit flies. Many studies show that this factor also contributes to lifespan extension in nematode *Caenorhabditis elegans* (Blackwell et al. 2015; Pitoniak and Bohmann 2015).

### Resistance of NDX-expressing fruit flies to organic toxicants

Our current data suggest that activation of Nrf2 may be a more general cause of prolonged lifespan in model organisms expressing aNDH. The common feature of these models is their resistance to multiple stresses, especially to the influence of xenobiotics (Sanz et al. 2010; Bahadorani et al. 2010; Gospodaryov et al. 2014). The most frequently used xenobiotic tested was paraquat (Sanz et al. 2010; Bahadorani et al. 2010). However, paraquat is a well-known redox-cycling agent, so it is rather difficult to dissect between its pro-oxidant effects and other possible toxicity mechanisms. We used a broader spectrum of xenobiotics with redox-cycling (menadione, alloxan) and pronounced electrophile (2,4-D, catechol) properties. Flies expressing aNDH (NDX) from *C. intestinalis* were markedly resistant to menadione, alloxan and 2,4-D, while more sensitive to catechol. The higher resistance of NDX-expressing flies to alloxan and 2,4-D in the current study suggests Nrf2 pre-activation in their cells compared to control flies. Chlorinated hydrocarbons, such as herbicide 2,4-D and insecticide dichlorodiphenyltrichloroethane (DDT), are electrophiles. It was shown that the *Drosophila* homolog of Nrf2, the protein CncC, is constitutively active in fruit fly lines resistant to DDT (Misra et al. 2013). Another chlorinated hydrocarbon, chlorpromazine was shown to increase the expression of Nrf2 targets, such as genes coding for different cytochrome P450s and GST (Misra et al. 2011). Phenobarbital and caffeine, compounds structurally similar to alloxan, were also found to be activators of Nrf2 (Misra et al. 2011).

The sensitivity of NDX-expressing flies to catechol can be explained by interference of this compound with Nrf2 signalling. Catechol and its derivatives are potent activators of Nrf2 in mammals (Wang et al. 2010; Senger et al. 2016). However, catechol may exert its pro-oxidant properties in cells with pre-activated Nrf2 signalling. In particular, it has been shown that thiolated derivates of catechol-containing compounds, such as dopamine, may exhibit both antioxidant and pro-oxidant properties (Picklo et al. 1999). The appearance of these derivatives is especially possible in the case of pre-activated Nrf2, since this transcription factor regulates both the reduction and de novo synthesis of glutathione, a low molecular mass thiol-containing antioxidant (Lu 2013). The outcome, i.e. whether catechol exerts an anti- or pro-oxidant action, would likely depend on the presence of transition metal ions and specific types of ROS (Picklo et al. 1999; Wang et al. 2010).

### Resistance of NDX-expressing fruit flies to inorganic toxicants

Chromate and molybdate anions may influence several different systems of cellular antioxidant defence and signalling pathways simultaneously. There are several reports that chromium may induce Nrf2 targets as well as the c-Jun-N-terminal kinase (JNK) pathway (He et al. 2007; Myers 2012). Recently, we have found that both chromate and molybdate partially mimic the effects of insulin in the fruit fly (Rovenko et al. 2014; Perkhulyn et al. 2015). Mild down-regulation of insulin signalling led to lifespan extension in numerous studies (Giannakou et al. 2008; Lushchak and Gospodaryov 2017; Tatar et al. 2014; Bitto et al. 2015). This finding might imply that up-regulation of insulin signalling by insulin mimetics, such as chromate and molybdate, would shorten lifespan or attenuate the pro-longevity effect of the aNDH. Similarly to the situation we observed with catechol, Nrf2 signalling, pre-activated by NDX, may enhance the toxicity of chromate ions.

Another inorganic poison, used in the current study, is potassium iodate. Iodate is frequently used for the experimental induction of retinal degeneration. It was shown that activation of Nrf2 signalling helps preventing iodate-induced retinal damage (Sachdeva et al. 2014). We therefore hypothesize that hormetic pre-activation of Nrf2 by NDX may attenuate iodate toxicity in NDX-expressing flies.

### Activities of enzymes regulated by Nrf2

Prolonged lifespan, resistance to xenobiotics such as 2,4-D and to other stresses suggest Nrf2 as a possible mediator of the effects of NDX. The aNDH Ndi1p from the budding yeast *S. cerevisiae* was previously shown to promote ROS generation via reverse electron transfer through complex I of the conventional RC (Scialò et al. 2016). A similar effect of NDX could account for the activation of Nrf2 in NDX-expressing flies. To test this idea, we analysed specific Nrf2 targets, most of which are involved in detoxification of xenobiotics and ROS scavenging. These targets include the cytochromes P450, which hydroxylate foreign organic compounds and different isoenzymes of GST, which conjugate organic compounds with glutathione, facilitating their following decomposition and/or excretion (Misra et al. 2011; Bruns et al. 2015; Lewis et al. 2015; Lushchak and Gospodaryov 2017). The Nrf2 pathway helps insects to resist organic insecticides (Misra et al. 2011, 2013), and was previously found to be activated in response to inorganic toxicants like lithium (Castillo-Quan et al. 2016), leading to enhanced expression of isoenzymes GstD2, GstD6, and GstD10. GstD2 and GstD7 are also induced by phenobarbital and caffeine (Misra et al. 2011). These findings imply that GST may be one of the most pronounced, easily detected targets of Nrf2 in fruit flies. Accordingly, we detected enhanced GST activity in NDX-expressing flies. In other organisms, G6PDH, an enzyme associated with antioxidant defence, is up-regulated by Nrf2. Although this has not been demonstrated previously in *Drosophila*, we found the enzyme to be elevated in NDX-expressing males, but not females. The *D. melanogaster* gene *Zw* encoding G6PDH is located on the X chromosome and is dosage-overcompensated in males (Luckinbill et al. 1990). *Zw* over-expression has been shown to prolong lifespan (Orr et al. 2013), and flies selected for long lifespan were found to possess the more active allozyme B of G6PDH (Luckinbill et al. 1990). Genes coding for the NADP-linked malate and isocitrate dehydrogenases are also targets of Nrf2 in mammals (Misra et al. 2011; Holmström et al. 2016), although their activities were not affected by NDX expression in flies. Several splice variants of *CncC* mRNA are known (Pitoniak and Bohmann 2015), which may activate distinct subsets of Nrf2 targets in different metabolic and developmental contexts.

## Conclusions

Our current and previous data suggest that longevity is closely associated with resistance to stresses, including oxidative stress caused by xenobiotics. We observed previously that life-prolonging preparations were effective under all dietary regimens except for high-protein low-carbohydrate diets (Gospodaryov et al. 2013). Our current data show a similar interaction profile for a lifespan-extending genetic intervention. In addition, we show that fruit flies expressing aNDH have increased expression of some Nrf2 targets, namely glutathione *S*-transferase and glucose 6-phosphate dehydrogenase. This strengthens the idea that Nrf2/Keap1 signalling responsible for the resistance of organisms to xenobiotics can be linked to pathways regulating metabolism and energy production, such as TOR, insulin/FOXO, and AMPK pathways. A detailed examination of how these pathways mediate life-prolonging effects of medications and genetic interventions is an important goal for future studies. Mitochondria and ROS metabolism are common denominators of these signalling pathways (Fernández-Ayala et al. 2010; Murugaiyah and Mattson 2015; Sanz 2016; Holmström et al. 2016). The role of mitochondria in lifespan extension conferred by ROS-mediated hormesis will undoubtedly be a fruitful topic for further investigation.

## Electronic supplementary material

Below is the link to the electronic supplementary material.

Supplementary material 1 (PDF 989 kb)
