# Correction to: Alternative NADH dehydrogenase extends lifespan and increases resistance to xenobiotics in *Drosophila*

**DOI:** 10.1007/s10522-020-09858-y

**Published:** 2020-01-27

**Authors:** Dmytro V. Gospodaryov, Olha M. Strilbytska, Uliana V. Semaniuk, Natalia V. Perkhulyn, Bohdana M. Rovenko, Ihor S. Yurkevych, Ana G. Barata, Tobias P. Dick, Oleh V. Lushchak, Howard T. Jacobs

**Affiliations:** 1 Department of Biochemistry and Biotechnology, Vasyl Stefanyk Precarpathian National University, Ivano-Frankivsk, Ukraine; 2 Division of Redox Regulation, DKFZ-ZMBH Alliance, German Cancer Research Center (DKFZ), Heidelberg, Germany; 3 Faculty of Medicine and Health Technology, Tampere University, Tampere, Finland; 4 Present Address: Department of Biosciences, Institute of Biotechnology, University of Helsinki, Helsinki, Finland; 5 Department of Biochemistry and Biotechnology, Faculty of Natural Sciences, Vasyl Stefanyk Precarpathian National University, 57 Shevchenko Str., Ivano-Frankivsk, 76018 Ukraine

## Correction to: Biogerontology 10.1007/s10522-019-09849-8

The article Alternative NADH dehydrogenase extends lifespan and increases resistance to xenobiotics in *Drosophila*, written by Dmytro V. Gospodaryov, Olha M. Strilbytska, Uliana V. Semaniuk, Natalia V. Perkhulyn, Bohdana M. Rovenko, Ihor S. Yurkevych, Ana G. Barata, Tobias P. Dick, Oleh V. Lushchak and Howard T. Jacobs, was originally published electronically on the publisher’s internet portal on 20 November 2019 without open access. With the author(s)’ decision to opt for Open Choice the copyright of the article changed on 21 January 2020 to © The Author(s) 2020 and the article is forthwith distributed under a Creative Commons Attribution 4.0 International License (https://creativecommons.org/licenses/by/4.0/), which permits use, sharing, adaptation, distribution and reproduction in any medium or format, as long as you give appropriate credit to the original author(s) and the source, provide a link to the Creative Commons licence, and indicate if changes were made. The original article has been corrected.

