# Supplementary material for: Alternative NADH dehydrogenase extends lifespan and increases resistance to xenobiotics in Drosophila
Source: Biogerontology. 2019 Nov 20;21(2):155–71. doi: 10.1007/s10522-019-09849-8 (PMC7056681; doi:10.1007/s10522-019-09849-8)
Supplement: Supplementary file 1 — Supplementary material 1 (PDF 989 kb) [file 10522_2019_9849_MOESM1_ESM.pdf]

Supplementary material of

Alternative NADH dehydrogenase extends lifespan and increases resistance to xenobiotics in *Drosophila*

Dmytro V. Gospodaryov[1], Olha M. Strilbytska[1], Uliana V. Semaniuk[1], Natalia V. Perkhulyn[1], Bohdana M. Rovenko[1]*, Ihor S. Yurkevych[1], Ana G. Barata[2], Tobias P. Dick[2], Oleh V. Lushchak[1], Howard T. Jacobs[3]

[1]Department of Biochemistry and Biotechnology, Vasyl Stefanyk Precarpathian National University, Ivano-Frankivsk, Ukraine

[2]Division of Redox Regulation, DKFZ-ZMBH Alliance, German Cancer Research Center (DKFZ), Heidelberg, Germany

[3]Faculty of Medicine and Health Technology, Tampere University, Tampere, Finland

*Present address: Department of Biosciences, Institute of Biotechnology, University of Helsinki, Helsinki, Finland

Address correspondence to: Dmytro V. Gospodaryov, Department of Biochemistry and Biotechnology, Faculty of Natural Sciences, Vasyl Stefanyk Precarpathian National University, 57 Shevchenko Str., Ivano-Frankivsk, 76018, Ukraine. E-mail: dmytro.hospodarov@pu.if.ua

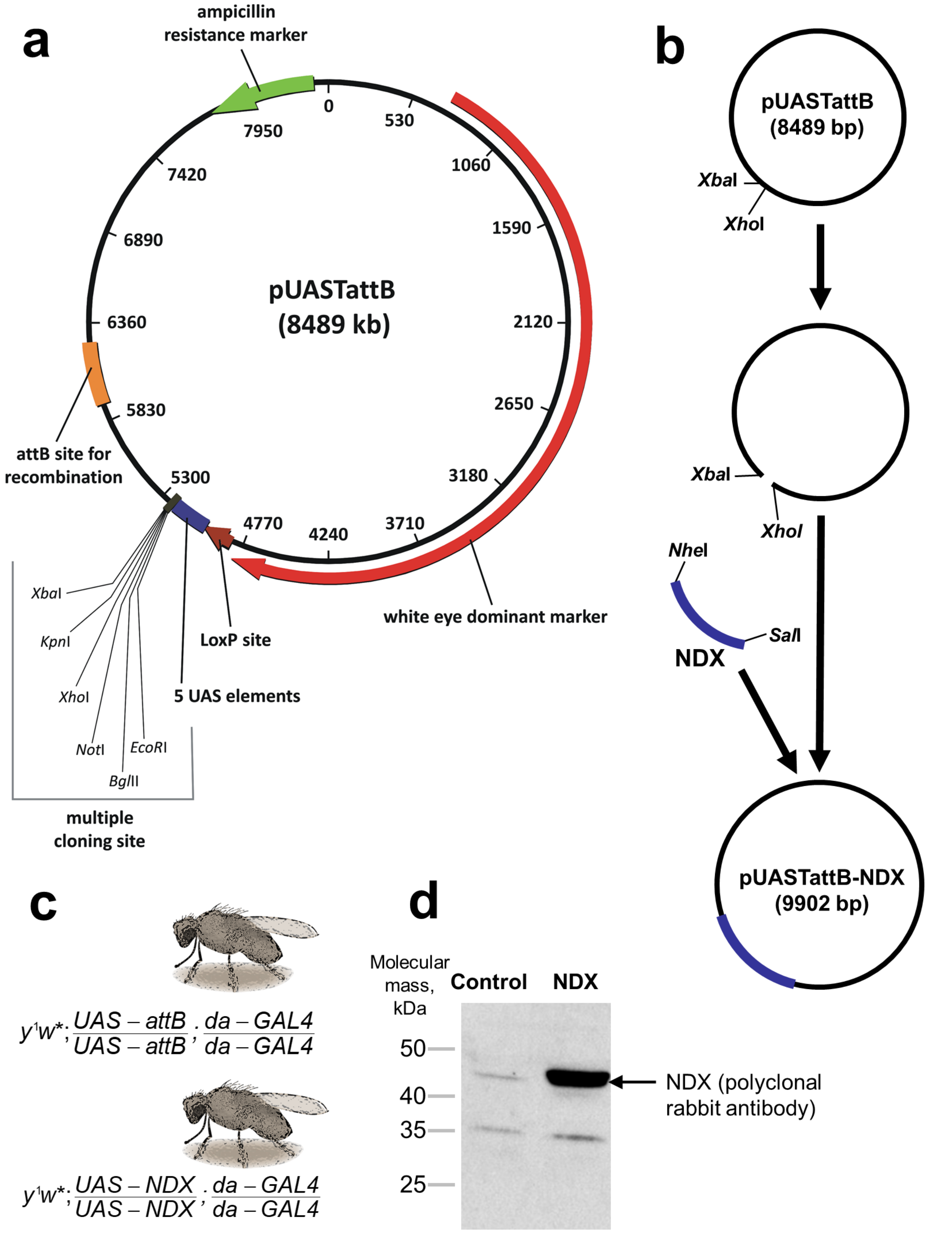

Fig. S1 Information on cloning strategy, fruit fly genotypes and confirmation of NDX expression in individuals of fruit fly lines used in the study (Gospodaryov et al. 2014). **a** Architecture of the cloning vector pUASTattB used for cloning NDX. **b** Strategy of NDX cloning. **c** Genotypes of control (top) and NDX-expressing lines (bottom). **d** Western blot confirming expression of NDX

Table S1 Prediction of NDX localization and its mitochondrial targeting sequence by different software

Software	Cleavage site	Score(% probability)	Organelle	Reference
MitoProt II	49|50	82.6	M	http://ihg.gsf.de/ihg/mitoprot.html
Targetp v1.1	24|25	29.2	M	http://www.cbs.dtu.dk/services/TargetP/
PSORT II:	–	43.5	M	http://psort.hgc.jp/form2.html
GvH	19|20	–		
Gavel	41|42	–		
WoLFPSORT	–	25.5	M	http://wolfpsort.seq.cbrc.jp/
iPSORT	29|30		M	http://ipsort.hgc.jp/
YLoc	–	97.3	M	http://abi.inf.uni-tuebingen.de/Services/YLoc/webloc.cgi
CELLO	–	–	M	http://cello.life.nctu.edu.tw/
Predotar	–	54.0	M	https://urgi.versailles.inra.fr/predotar/predotar.html
SherLoc	–	68.0	M	http://abi.inf.uni-tuebingen.de/Services/SherLoc/

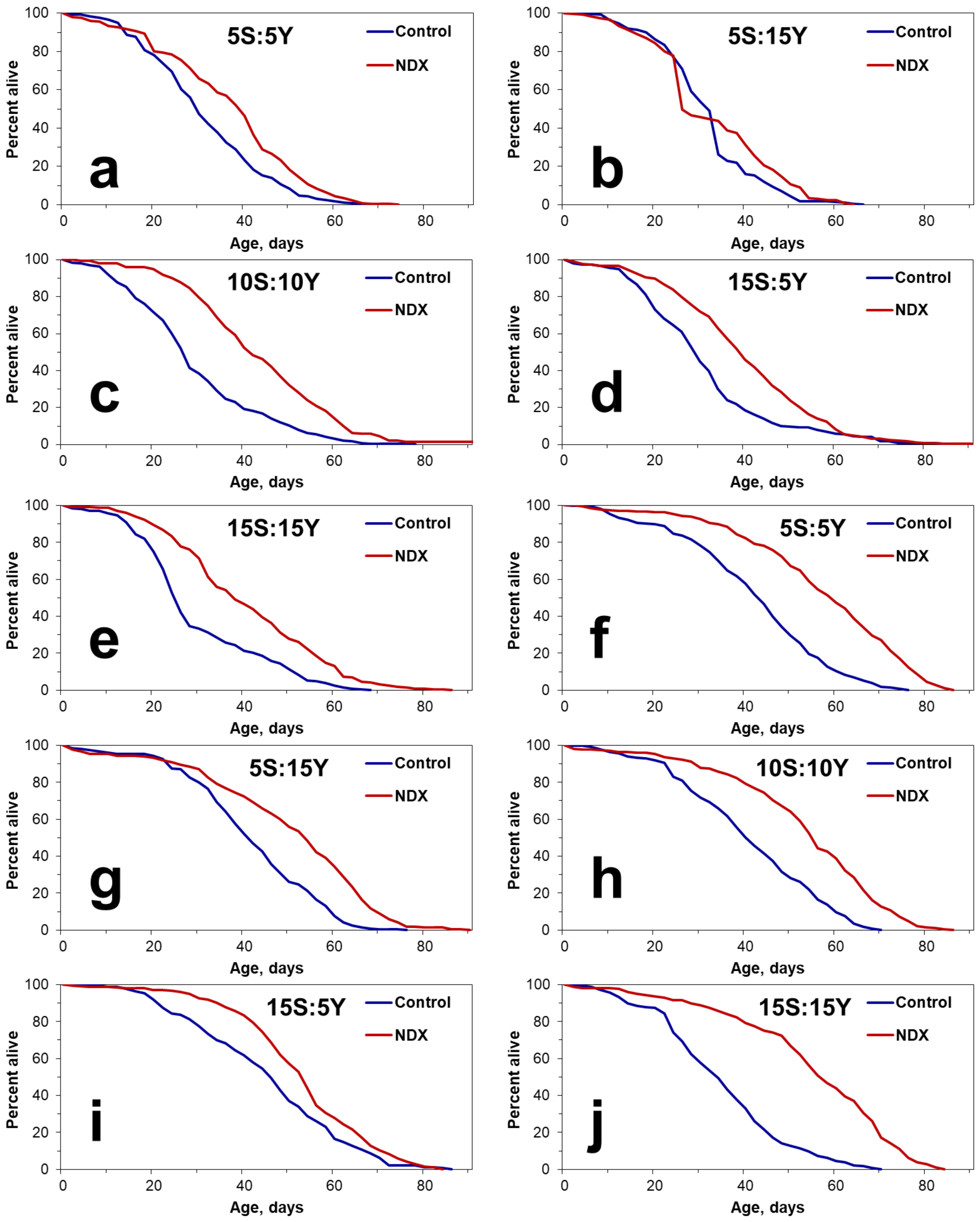

Fig. S2 Lifespan curves of the control ('Control') and NDX-expressing ('NDX') *Drosophila melanogaster* on the diets containing sucrose (S) and yeast (Y). The percentages of dietary components are shown inside plots. From 221 to 302 individuals were used per line and diet. Panels **a**-**e** are lifespans for male cohorts, panels **f**-**j** are for female cohorts.

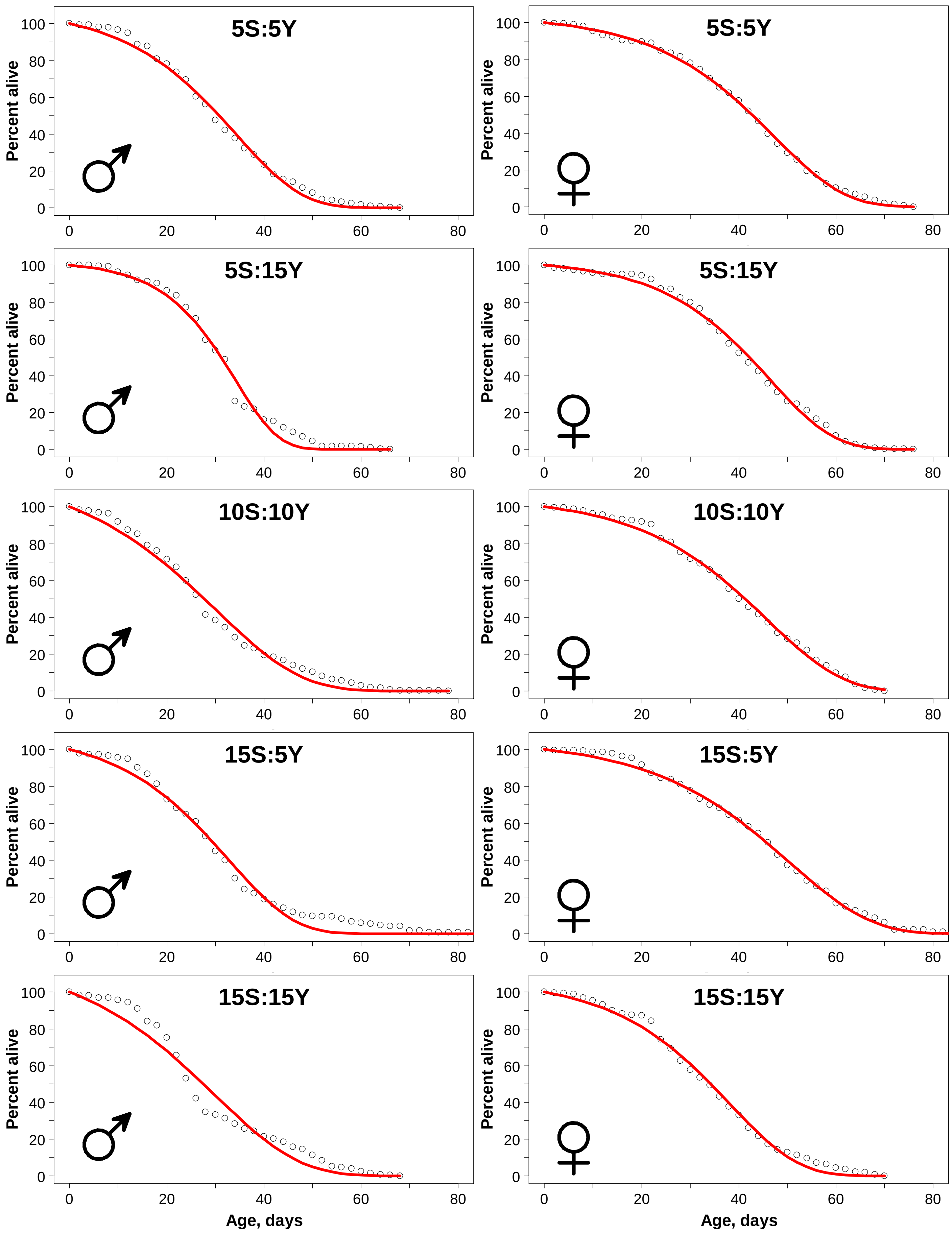

Fig. S3 Approximation (red lines) of lifespan curves for the control line on different diets by modified Gompertz equation $N_t = N_0 \cdot \exp\{A \cdot (1 - \exp(\alpha t))/\alpha t\}$ where N_t is the number of alive individuals at any time moment, N_0 is an initial cohort size, and A and α are age-independent and age-dependent parameters of Gompertz equation, respectively

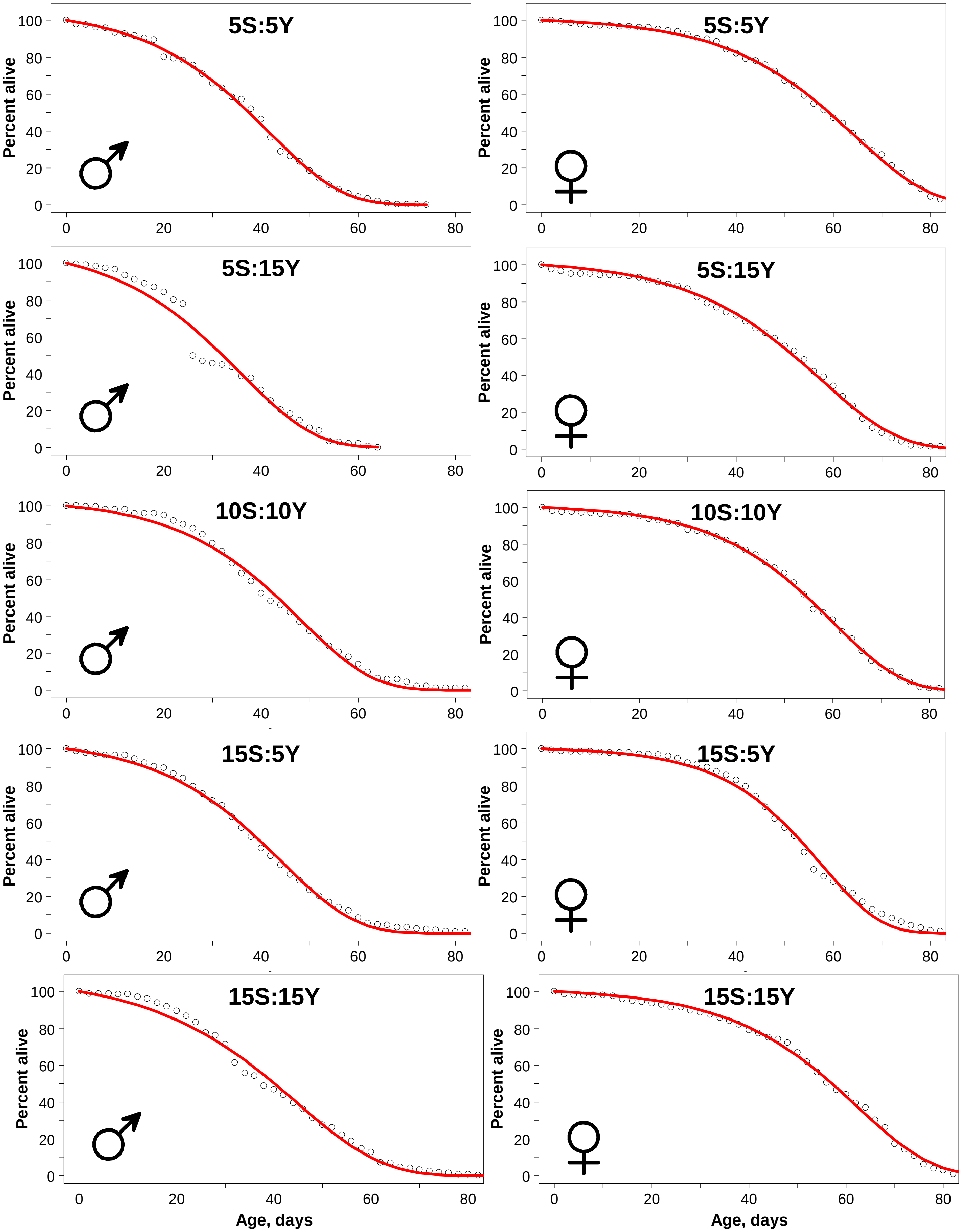

Fig. S4 Approximation (red lines) of lifespan curves for the NDX-expressing line on different diets by modified Gompertz equation $N_t = N_0 \cdot \exp\{A \cdot (1 - \exp(\alpha t)) / \alpha t\}$ where N_t is the number of alive individuals at any time moment, N_0 is an initial cohort size, and A and α are age-independent and age-dependent parameters of Gompertz equation, respectively

Table S2 Four-way analysis of variance of resistance of control and NDX-expressing flies to different concentrations of sodium chromate, using following factors: fruit fly line, age, sex, and salt concentration

Factor	Degrees of freedom	Sum of Squares	Mean Square	*F*-value	*p*-value	Significance of the main effect or interaction
Line	1	9241	9241	140.22	0.0000000	***
Age	2	24068	12034	182.599	0.0000000	***
Sex	1	55198	55198	837.563	0.0000000	***
Concentration	2	58713	29356	445.446	0.0000000	***
Line×Age	2	683	342	5.185	0.0067330	**
Line×Sex	1	410	410	6.227	0.0137510	*
Age×Sex	2	1939	970	14.711	0.0000016	***
Line×Concentration	2	1112	556	8.439	0.0003470	***
Age×Concentration	4	2807	702	10.648	0.0000001	***
Sex×Concentration	2	3507	1753	26.607	0.0000000	***
Line×Age×Sex	2	493	246	3.738	0.0262300	*
Line×Age×Concentration	4	92	23	0.349	0.8445880	
Line×Sex×Concentration	2	3	1	0.02	0.9805350	
Age×Sex×Concentration	4	744	186	2.821	0.0273980	*
Line×Age×Sex×Concentration	4	335	84	1.271	0.2843250	
Residuals	139	9161	66			

Table S3 Four-way analysis of variance of resistance of control and NDX-expressing flies to different concentrations of sodium molybdate, using the same factors as in Table S2

Factor	Degrees of freedom	Sum of Squares	Mean Square	*F*-value	*p*-value	Significance of the main effect or interaction
Line	1	43269	43269	109.243	0.0000000	***
Age	2	505	253	0.638	0.5298900	
Sex	1	130714	130714	330.019	0.0000000	***
Concentration	2	1346614	673307	1699.923	0.0000000	***
Line×Age	2	8837	4418	11.155	0.0000292	***
Line×Sex	1	764	764	1.93	0.1666800	
Age×Sex	2	155	77	0.195	0.8228600	
Line×Concentration	2	51718	25859	65.287	0.0000000	***
Age×Concentration	4	3988	997	2.517	0.0434600	*
Sex×Concentration	2	56695	28347	71.57	0.0000000	***
Line×Age×Sex	2	2924	1462	3.691	0.0270900	*
Line×Age×Concentration	4	30569	7642	19.294	0.0000000	***
Line×Sex×Concentration	2	4406	2203	5.562	0.0046200	**
Age×Sex×Concentration	4	2206	552	1.393	0.2388600	
Line×Age×Sex×Concentration	4	2918	730	1.842	0.1233800	
Residuals	160	63373	396			
